# Formulation Optimization of No Added Sugar Cookies Supplemented with Rice Bran Powder and Elderberry Powder

**DOI:** 10.3390/foods15152584

**Published:** 2026-07-23

**Authors:** Shimin Wang, Ziyang Ma, Longda Qi, Wenfan Bian, Shurong Xin, Yan Shi, Xiulin Wang

**Affiliations:** 1 College of Agronomy, Qingdao Agricultural University, Qingdao 266109, China; 20230202704@stu.qau.edu.cn (S.W.); 20230203990@stu.qau.edu.cn (Z.M.); 20230210098@stu.qau.edu.cn (L.Q.); yanshi@qau.edu.cn (Y.S.); 2 Institute of Agricultural Resource and Environment, Shandong Academy of Agricultural Sciences, Jinan 250100, China; gxbwf@163.com (W.B.); xinshurong@126.com (S.X.)

**Keywords:** no added sugar cookies, rice bran powder, elderberry powder, response surface methodology, formulation optimization

## Abstract

Driven by the growing demand for nutritious no added sugar bakery products, this study developed no added sugar cookies supplemented with rice bran powder, elderberry powder, and natural sweeteners. Single-factor experiments and response surface methodology (Box–Behnken design) were used to optimize the formulation. The established quadratic model was highly significant (*p* < 0.0001, *R*^2^ = 0.9870). Rice bran powder exhibited the dominant influence on sensory quality, followed by mogrosides, elderberry powder, and butter. The optimal practical formulation was determined to be 8 g rice bran powder, 3 g elderberry powder, 91 g butter, 0.3 g mogrosides, together with 92 g low-gluten flour, 2 g fructooligosaccharides (FOS), 0.02 g steviol glycosides, 30 g beaten egg and 20 g milk. The optimized cookies possessed a uniform pale-pink color, a crisp texture, and a balanced aroma combining rice bran and elderberry characteristics. Compared with commercial products, the optimized cookies contained higher contents of protein and total dietary fiber, lower levels of carbohydrates and sodium, as well as abundant bioactive components, including total polyphenols (132.8 ± 0.85 mg/100 g), flavonoids (62.77 ± 0.87 mg/100 g), and anthocyanins (23.03 ± 0.99 mg/100 g). These results provide a scientific basis and technical guidance for the formulation and industrial application of no added sugar functional cookies and further expand the application potential of rice bran as a value-added by-product of rice processing.

## 1. Introduction

Continuous advances in science and technology, along with improvements in living standards, have increased consumer awareness of the relationship between diet and health and demand for nutritious and health-oriented food products. Cookies are among the most widely consumed convenience foods. They are traditionally made from soft wheat flour, sugar, and fat, with minor ingredients such as milk, salt, flavoring agents, and aerating agents [1]. Their production generally involves cold dough preparation, shaping processes (e.g., extrusion, molding, wire cutting, or roller imprinting), and baking, resulting in products with a crisp texture and desirable sensory characteristics. However, conventional cookies are typically high in sugar and low in protein [2]. Excessive consumption of such products has been associated with an increased risk of obesity, diabetes, and cardiovascular diseases, which contrasts with current dietary recommendations emphasizing balanced, health-promoting food choices. As a result, the development of no added sugar, nutrient-dense cookies has become a prominent area of research in the food industry.

Rice bran is a major by-product of rice milling and primarily comprises the outer layers of rice grains, including the pericarp, seed coat, outer endosperm, aleurone layer, and embryo [3]. It is a rich source of dietary fiber, protein, lipids, vitamins, minerals, and bioactive compounds, including phenolic acids, flavonoids, anthocyanins, γ-oryzanol, and phytosterols. These components have been reported to provide various health benefits, including cholesterol reduction, blood glucose regulation, enhanced intestinal motility, and cancer prevention [4]. Particularly, soluble and insoluble dietary fibers from rice bran contribute to improved gut health by promoting the growth of beneficial microorganisms while suppressing harmful bacterial populations [5,6]. Moreover, phenolic extracts from rice bran demonstrate antioxidant and anti-inflammatory activities, indicating potential applications in the prevention and management of chronic oxidative stress-related conditions [7]. A wide range of bioactive components further supports the chemopreventive, cardioprotective, hepatoprotective, immunomodulatory, neuroprotective, and lipid-lowering properties of rice bran [8]. Despite its high nutritional and functional value, utilization of rice bran in food applications remains limited due to inadequate consumer awareness and technical challenges related to processing and stabilization [9]. Therefore, incorporating rice bran into cookie formulations is an effective approach to improving nutritional quality while enhancing the value-added use of agricultural by-products.

Elderberry (*Sambucus nigra* L.) is recognized as a nutritionally rich fruit containing organic acids, vitamins, proteins, anthocyanins, polyphenols, flavonoids, and other bioactive compounds [10]. Elderberry powder, made by drying fresh elderberries, preserves most of the nutritional and functional components while providing improved storage stability and processing convenience. Numerous studies have shown that elderberry exhibits antioxidant, anti-inflammatory, immunomodulatory, and antiviral activities. Accordingly, elderberry powder has been increasingly incorporated into the development of health-promoting foods, including beverages, bakery products, and dietary supplements [11]. Studies have demonstrated that the supplementation of elderberry powder in cookie formulations can effectively increase the total polyphenol and total flavonoid contents of cookies, markedly enhance their antioxidant capacity and functional properties, and thus substantially improve the nutritional value of traditional cookies [12]. However, the interaction effects between elderberry powder and other functional ingredients in cookie systems have not been systematically investigated.

Sugar plays a crucial role in normal cookie formulations by contributing sweetness, texture, and shelf-life stability [13]. However, excessive consumption of sucrose, the primary sweetening agent in cookies, has been associated with an increased risk of obesity, type 2 diabetes, and dental caries [14]. Higher health concerns associated with high sugar intake have therefore intensified interest in natural, low-calorie alternatives to sucrose. Fructooligosaccharides (FOS), steviol glycosides, and mogrosides represent naturally derived functional sweeteners that have attracted increasing attention in food applications [15,16]. As a functional oligosaccharide, FOS resists digestion and absorption in the human small intestine and can be selectively fermented by beneficial gut microbiota, such as bifidobacteria, contributing to intestinal health and microbial balance [15]. Steviol glycosides, extracted from *Stevia rebaudiana* leaves, provide high sweetness intensity with negligible caloric value and exhibit favorable biological properties, including reactive oxygen species (ROS) scavenging activity, potential modulation of blood glucose and blood pressure, and protective effects against metabolic syndrome and atherosclerosis [17]. Mogrosides, isolated from monk fruit (*Siraitia grosvenorii*), are high-potency, near-zero-calorie sweeteners that also show antioxidant, metabolic regulatory, and lung-moistening activities [18]. As natural food additives, mogrosides have been widely applied in a variety of food products, including beverages, dairy products, condiments, and baked goods [19]. To date, few studies have systematically investigated how mogroside dosage interacts with rice bran and elderberry powders to affect the comprehensive quality of no added sugar cookies.

To address the aforementioned research gaps, this study employed a composite low-calorie sweetener system to replace conventional sucrose. The dosages of FOS (2 g per 100 g base flour) and steviol glycosides (0.02 g per 100 g base flour) were set below the typical dosage ranges used in no added sugar bakery formulations. This lower baseline was deliberately chosen to create formulation headroom for mogrosides, allowing mogrosides to serve as the primary variable for adjusting overall sweetness and for balancing the undesirable off-flavors contributed by rice bran and elderberry powders. Accordingly, rice bran powder, low-gluten flour, and elderberry powder were integrated as the main raw materials, and a fixed combination of FOS and steviol glycosides together with variable mogrosides replaced refined sucrose to develop nutritionally improved no added sugar cookies. The specific objectives of this study were to (i) evaluate the effects of different addition levels of rice bran powder, elderberry powder, butter, and mogrosides on the physicochemical, textural and sensory properties of cookies through single-factor experiments; (ii) optimize the formulation using response surface methodology and identify the optimal addition ranges of these factors; and (iii) provide a scientific basis for the development of functional, no added sugar bakery products that utilize agricultural by-products. This work also aims to achieve the dual goals of food nutritional improvement and the high-value utilization of agricultural by-products, providing feasible technical references for the green and healthy development of the bakery industry.

## 2. Materials and Methods

### 2.1. Materials

Low-gluten flour was purchased from Xinxiang Liangrun Whole Grain Food Co., Ltd. (Xinxiang, China). Rice bran was collected from the milling of grains of the japonica rice cultivar Shengdao 20. The rice bran was spread in a thin layer and dried in an oven at 120 °C for 20 min according to Lv et al. [20]. After cooling to room temperature, the samples were pulverized for 8 min by a vibrating ultrafine pulverizer (SYFM-8I, Jinan Songyue Machinery Co., Ltd., Jinan, China), with cooling water circulated continuously throughout the process. Subsequently, the pulverized samples were sieved through a 100-mesh sieve to prepare rice bran powder with a particle size of ≤150 μm. Elderberry powder (purity ≥ 99%) was purchased from Shaanxi Junhe Biotechnology Co., Ltd. (Xi’an, China). Butter (President brand, imported from France) was purchased from Lactalis Trading (Shanghai) Co., Ltd. (Shanghai, China). FOS (purity ≥ 95%) was obtained from Jiangmen DeAng Biotechnology Co., Ltd. (Jiangmen, China). Steviol glycosides (purity ≥ 90%) were purchased from Shandong Haigen Biotechnology Co., Ltd. (Jining, China). Mogrosides (mogroside V 50%, total mogrosides 98%) were acquired from Xi’an Baiwangda Pharmaceutical Technology Co., Ltd. (Xi’an, China). Whole milk was purchased from Inner Mongolia Yili Industrial Group Co., Ltd. (Hohhot, China). Fresh eggs were purchased from a local market. Commercial control product 1 (CP1 ingredients: wheat flour, white sugar, butter, vegetable oil (contains vitamin E), glucose syrup, eggs, desiccated coconut, seedless raisins, skimmed milk powder, salt, ammonium bicarbonate, food flavoring) was purchased from Mayora Food (Shanghai) Co., Ltd. (Shanghai, China). Commercial control product 2 (CP2 ingredients: wheat flour, baking cream [vegetable oil (refined vegetable oil, vitamin E), water, anhydrous milk fat, salt, food additives (mono- and diglycerides of fatty acids, phospholipids, potassium sorbate, citric acid, β-carotene), food flavoring], white sugar, butter (≥1.5%), edible corn starch, cheese powder (1.3%), salt, food flavoring) was purchased from Dongguan Fengxi Food Co., Ltd. (Dongguan, China).

### 2.2. Experimental Methods

#### 2.2.1. Basic Recipe

The basic formulation of the no added sugar cookies was based on weight. As shown in Table 1, rice bran powder, elderberry powder, butter, and mogrosides were selected as experimental variables, with 5 gradient levels set for each variable. The addition amounts of fructooligosaccharides (FOS), steviol glycosides, beaten egg and whole milk were fixed at constant values in all formulations. The total amount of low-gluten flour and rice bran powder was kept constant at 100.00 g across all formulations. All ingredients were weighed using an electronic balance (AR2140, OHAUS Instruments (Shanghai) Co., Ltd., Shanghai, China).

#### 2.2.2. Preparation Method

No added sugar cookies supplemented with rice bran powder and elderberry powder were prepared as follows. Butter was softened at room temperature until gentle finger pressure produced a slight indentation. FOS, steviol glycosides, and mogrosides were premixed for uniform dispersion. The blended sweetener mixture was then added to the softened butter and whipped with an electric mixer (HM900, Philips (China) Investment Co., Ltd., Shanghai, China) until a light, fluffy consistency with increased volume was achieved. Beaten egg was gradually added to the butter mixture in two to three portions, with thorough mixing after each addition to ensure complete emulsification and prevent oil–water separation. Whole milk was then added and mixed until a homogeneous mixture was formed.

Low-gluten flour, rice bran powder, and elderberry powder were passed through a 100-mesh sieve and gently folded into the wet mixture with a spatula until no visible dry material remained. Excessive mixing was avoided to limit gluten development and preserve cookie texture. The resulting dough was transferred to a piping bag and extruded onto baking trays to form uniform cookie blanks with appropriate spacing. Baking was carried out in a preheated electric oven (F40, Guangdong Weishida Electric Technology Co., Ltd., Foshan, China) at 160 °C for 5 min to set the shape, followed by baking at 140 °C for 20–25 min until slight browning was observed at the edges. Baking time was adjusted based on oven performance. After baking, cookies were allowed to cool on the tray for 5–10 min, then transferred to a cooling rack and left to cool to room temperature.

### 2.3. Single-Factor Experiments

Single-factor experiments were performed to evaluate the effects of rice bran powder, elderberry powder, butter, and mogrosides on the sensory quality of no added sugar cookies supplemented with rice bran and elderberry powders. These experiments were conducted to determine the appropriate ranges of variation for each factor before response surface optimization.

#### 2.3.1. Effect of Rice Bran Powder Addition on the Sensory Quality of Cookies

The addition range of rice bran powder was determined according to He et al. [21], who optimized the processing technology of sea rice bran biscuits and found that the biscuit obtained the highest sensory score at a rice bran powder addition of 10.53% (based on 100% total flour weight). Based on this optimum, the addition amounts of rice bran powder were varied at 6.00, 8.00, 10.00, 12.00 and 14.00 g, while the corresponding low-gluten flour was adjusted to 94.00, 92.00, 90.00, 88.00 and 86.00 g, respectively, to maintain a constant total mass of 100.00 g for the rice bran powder and low-gluten flour combination. The remaining ingredients were fixed at 3.00 g elderberry powder, 80.00 g butter, 0.300 g mogrosides, 2.000 g FOS, 0.020 g steviol glycosides, 30.00 g beaten egg, and 20.00 g whole milk. Cookie samples were prepared following the procedure described above, and sensory evaluation was conducted to assess the influence of rice bran powder addition on overall sensory scores.

#### 2.3.2. Effect of Elderberry Powder Addition on the Sensory Quality of Cookies

Referring to the findings of Hlavácová et al. [22], who supplemented Linz biscuits with 3% elderberry fruit powder by product mass in their research, five gradient addition levels of elderberry powder were set in this experiment to investigate its effects on the sensory quality of cookies, with the addition amounts of 1.00 g, 2.00 g, 3.00 g, 4.00 g and 5.00 g, respectively. During these additions, the remaining formulation components were fixed at 90.00 g low-gluten flour, 10.00 g rice bran powder, 80.00 g butter, 0.300 g mogrosides, 2.000 g FOS, 0.020 g steviol glycosides, 30.00 g beaten egg, and 20.00 g whole milk. Cookie samples were prepared following the procedure described in Section 2.2.2, and sensory evaluation was conducted to determine the effect of elderberry powder addition on overall sensory scores.

#### 2.3.3. Effect of Butter Addition on the Sensory Quality of Cookies

To assess the impact of butter content on sensory attributes, butter was added at levels of 60.00, 70.00, 80.00, 90.00, and 100.00 g according to Chai et al. [23]. All other ingredients were maintained at constant levels, including 90.00 g low-gluten flour, 10.00 g rice bran powder, 3.00 g elderberry powder, 0.300 g mogrosides, 2.000 g FOS, 0.020 g steviol glycosides, 30.00 g beaten egg, and 20.00 g whole milk. Cookies were produced using the standardized preparation method and examined for sensory quality.

#### 2.3.4. Effect of Mogroside Addition on the Sensory Quality of Cookies

Based on preliminary experiment results, five gradient addition levels of mogrosides (0.100, 0.200, 0.300, 0.400 and 0.500 g) were set to explore their effects on the sensory quality of cookies. The other formulation included 90.00 g low-gluten flour, 10.00 g rice bran powder, 3.00 g elderberry powder, 80.00 g butter, 2.000 g FOS, 0.020 g steviol glycosides, 30.00 g beaten egg, and 20.00 g whole milk. Cookie preparation and sensory evaluation were carried out as described in the section above.

#### 2.3.5. Determination of Physicochemical and Textural Properties of Cookies

Water activity (*a_w_*) of cookies was measured at 25 ± 1 °C using a water activity meter (HD-3A, Wuxi Huake Instrument Co., Ltd., Wuxi, China). Color parameters (L*, a*, b*) were determined using a colorimeter (CR-400, Konica Minolta (China) Investment Ltd., Shanghai, China). Prior to testing, the instrument was calibrated with a standard white reference plate under CIE standard illuminant D65 with a 2° standard observer angle, and an 8 mm diameter measurement aperture was adopted. To guarantee representative measurement data, all color readings were collected on the top surface of each cookie, with cracked and edge regions excluded intentionally. Three independent cookie specimens were tested for each treatment group. Three random separate positions were measured on every single specimen, and the final color parameters of each group were calculated as the average of nine measured values.

Texture profile analysis (TPA) was performed using a texture analyzer (TMS-TOUCH, Food Technology Corporation (FTC), Sterling, VA, USA) equipped with a P/36R cylindrical probe. The test conditions were as follows: pre-test speed 2.0 mm/s, test speed 1.0 mm/s, post-test speed 1.0 mm/s, compression strain 30%, and time interval 5 s. Hardness (N), cohesiveness, springiness (mm), gumminess (N), and chewiness (mJ) were automatically calculated from the force–time curve. Hardness (N) is defined as the peak compressive force required to deform the sample to the specified strain (30%). Cohesiveness is the ratio of the positive force work area during the second compression to that during the first compression, representing the internal strength of the sample structure against deformation. Springiness (mm) is the distance the sample recovers elastically after the first compression, indicating its elastic recovery capacity. Gumminess (N) is calculated as hardness multiplied by cohesiveness, which reflects the force needed to break down the food matrix during mastication. Chewiness (mJ) is calculated as gumminess multiplied by springiness, representing the energy required to masticate the solid food until it can be swallowed. Each treatment was measured in five replicates, and results were expressed as mean ± standard deviation (SD).

### 2.4. Response Surface Optimization Experiment

Based on single-factor experimental results, four variables showing significant effects on cookie sensory quality were selected as independent factors: rice bran powder addition (A), elderberry powder addition (B), butter addition (C), and mogroside addition (D). The overall sensory score served as the response variable (Y). A Box–Behnken design (BBD) incorporating three coded levels (−1, 0, and 1) was used to optimize the formulation. The experimental design was established and analyzed using Design-Expert 13.0 software. A total of 29 experimental runs were arranged in the BBD test, including 24 factorial points and 5 central replicate points, and all experimental groups were prepared in two independent batches for sensory evaluation. The coded levels and corresponding actual values of the independent variables are shown in Table 2.

### 2.5. Sensory Evaluation of Cookies

Sensory evaluation was conducted by a trained panel of 10 panelists (5 males and 5 females, aged 22–45 years). The panel size was consistent with previous studies that have demonstrated that a trained panel of 8 to 10 assessors can provide reliable and reproducible sensory data when rigorous selection and training procedures are followed [24,25]. All panelists were nonsmokers with normal taste and odor acuity and were recruited following the general principles outlined in ISO 8586:2023 for the selection and training of sensory assessors [26]. Panelist selection involved an initial screening to confirm the absence of olfactory or gustatory impairments, as well as the ability to discriminate basic tastes (sweet, sour, salty, bitter) and recognize common food odors. Following selection, panelists completed a formal training program consisting of a series of standardization sessions, during which they were familiarized with the cookie-specific attributes evaluated in this study, including color, shape, texture, mouthfeel, and flavor. Attribute definitions, reference standards, and intensity scales were established using the consensus approach recommended for descriptive analysis panels [26]. The sensory panel was further validated according to ISO 11132:2021, which provides guidelines for assessing the overall performance of a quantitative descriptive panel and the performance of each panel member, applicable to the validation of training of individual assessors or panels and the performance monitoring of established panels [27].

Evaluations were conducted between 20 min and 2 h after the cookies cooled to room temperature in a dedicated sensory evaluation room under controlled conditions—standardized white lighting, ambient temperature between 20 and 22 °C, and a neutral odor environment—with panelists seated at least 1 m apart and instructed not to communicate during the evaluation to minimize cross-influence. Each sample was coded with a random three-digit code, and the presentation order of samples was fully randomized across panelists to minimize order and carryover effects. Panelists were blinded to the sample formulations and processing conditions. Two independent batches of each cookie sample were prepared for duplicate sensory testing, and the two replicates were evaluated on separate days. All 10 panelists scored every batch independently. The sensory evaluation was conducted according to Xiao et al. [28] and Chai et al. [23], with appropriate modifications tailored to the characteristics of cookies in this study. The sensory attributes observed included color (15 points), shape (15 points), texture (20 points), mouthfeel (20 points), and flavor (30 points), yielding a maximum total score of 100 points. Detailed scoring criteria for each attribute are presented in Table 3. Each cookie sample was independently evaluated by all 10 panelists, and the final sensory score was calculated as the mean of the individual panelist scores across both replicates.

### 2.6. Control Sample Preparation

A control cookie (CK) formulation was prepared without the addition of rice bran powder or elderberry powder. The formulation consisted of 100.00 g low-gluten flour, 91.00 g butter, 2.000 g FOS, 0.020 g steviol glycosides, 0.300 g mogrosides, 30.00 g beaten egg, and 20.00 g whole milk. The control cookies were produced following the same preparation and baking procedure described for the no added sugar cookies supplemented with rice bran and elderberry powders.

### 2.7. Nutritional Composition and Total Dietary Fiber Analysis

Nutritional composition and total dietary fiber content were analyzed by Qingdao Yuanxin Testing Technology Co., Ltd. (Qingdao, China). Energy content was determined according to GB 28050-2011 (National Food Safety Standard of the People’s Republic of China) using standard energy conversion coefficients of major nutritional components [29]. Protein content was measured using GB 5009.5-2016, Method 1 [30]. Organic nitrogen in samples was converted and quantified via digestion, distillation and titration to calculate protein content. Fat content was measured by Soxhlet extraction method (Method 1) according to GB 5009.6-2016; lipids were extracted from dried samples using organic solvents and quantified gravimetrically [31]. Carbohydrate content was analyzed in accordance with GB 28050-2011 [29]. The total carbohydrate content was calculated by subtracting the contents of other measured components from the total sample mass. Sodium content was detected by flame atomic absorption spectrophotometry (Method 1) specified in GB 5009.91-2017 [32]. Sodium content was quantitatively determined based on the characteristic atomic absorption intensity of the sample solution in flame. Moisture and ash contents were measured in accordance with GB 5009.3-2016 (Method 1) [33] and GB 5009.4-2016 (Method 1) [34], respectively. Moisture content was determined by measuring the mass loss of samples after constant-temperature drying. Samples were ignited at high temperature to remove organics, and ash content was determined by constant-weight gravimetry. Total dietary fiber content was determined in accordance with GB 5009.88-2023 [35]. Total dietary fiber was determined by enzymatic hydrolysis to remove starch and protein, followed by gravimetric quantification.

### 2.8. Determination of Total Polyphenol, Flavonoid and Anthocyanin Contents

Total polyphenol content was measured by the Folin–Ciocalteu method as described by Wan et al. [36]. Total flavonoid content was determined by Qingdao Standard Testing Co., Ltd. (Qingdao, China) following the industry standard SN/T 4592-2016 (Determination of total flavonoids in export food products) [37]. Briefly, the method relies on the complexation reaction between flavonoids and aluminum salts under alkaline conditions to form stable yellow chromogenic complexes. The absorbance of the sample solution and rutin standard series solutions is measured at 420 nm using a spectrophotometer, and the total flavonoid content is quantified based on the established standard curve. Total anthocyanin content was measured using the pH differential method [38].

### 2.9. Statistical Analysis

Data obtained from single-factor experiments were processed using Microsoft Excel 2021, and graphical representations were generated using SigmaPlot 12.5. Response surface optimization data were analyzed using Design-Expert 13.0 to establish quadratic regression models and conduct analysis of variance (ANOVA). Duncan’s multiple range test was performed using SPSS 22.0 to evaluate significant differences among treatments, with statistical significance defined at *p* < 0.05.

## 3. Results and Discussion

### 3.1. Single-Factor Experimental Analysis

#### 3.1.1. Effect of Rice Bran Powder Addition on Physicochemical Properties, Textural Properties, and Sensory Scores of Cookies

**Figure 1:**
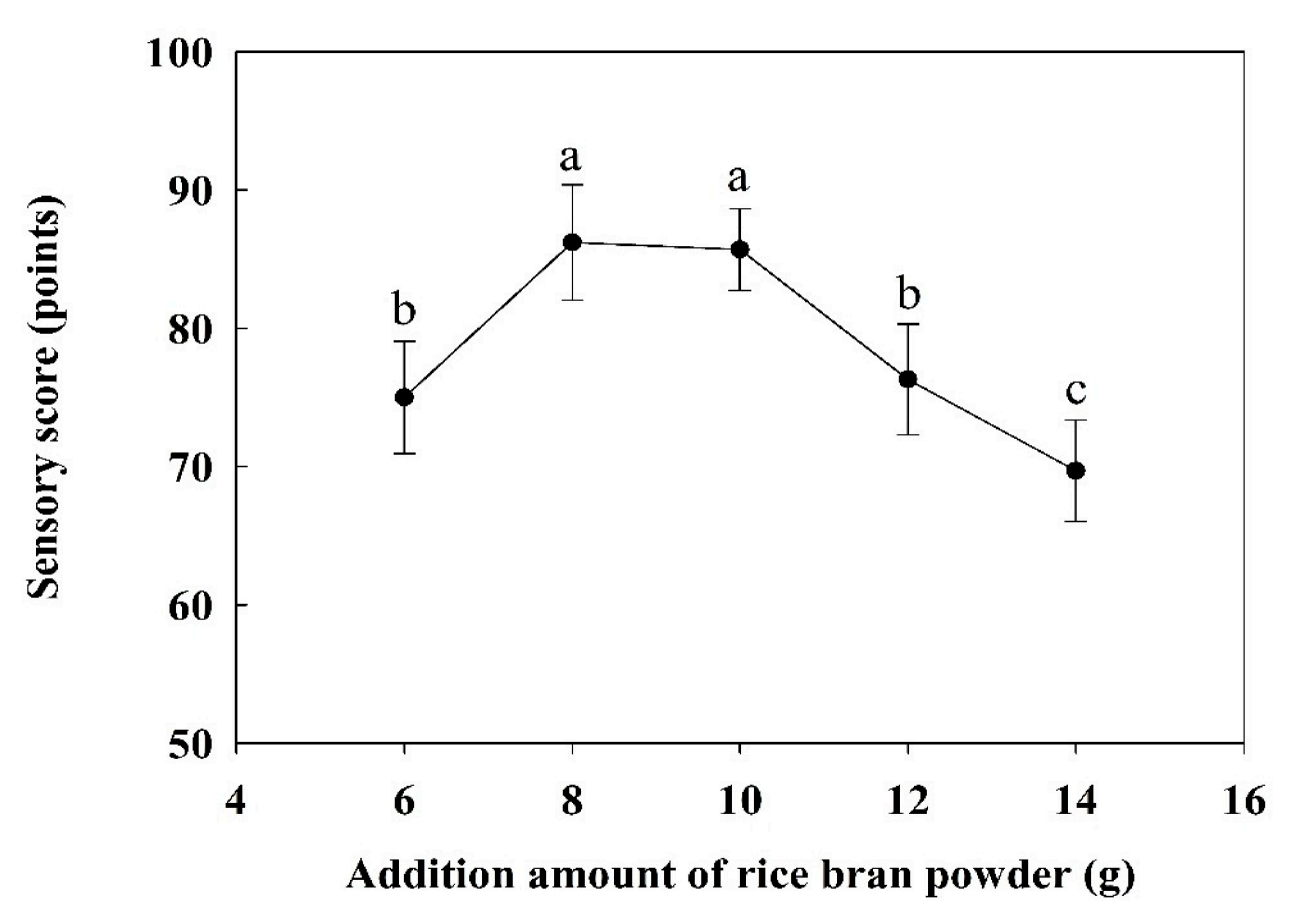
Effect of rice bran powder addition amount on the sensory score of cookies. Different lowercase letters indicate significant differences at *p* < 0.05.

Rice bran was thermally stabilized and finely ground to a particle size of ≤150 μm to improve its physicochemical functionality. Fine grinding is known to reduce particle size, increase specific surface area, and enhance dough solubility and dispersibility, thus facilitating nutrient bioavailability [39]. The physicochemical, textural, and sensory characteristics of the cookies were significantly influenced by the addition level of the rice bran powder (Table 4, Figure 1).

With the increase in rice bran powder addition, the water activity (*a_w_*) of cookies showed an overall significant decreasing trend from 0.504 to 0.423 (*p* < 0.05, Table 4). Although the *a_w_* values of all samples were below 0.6, a threshold sufficient to suppress most microbial proliferation, the further reduction in water activity can limit non-microbial quality deterioration, including the Maillard reaction and texture hardening during storage. This was closely related to the strong water-holding capacity of dietary fiber in rice bran, which could bind free water in the system and reduce water activity, thereby helping to extend the shelf life of the product [40]. In terms of color, the L* value declined significantly from 51.26 to 42.83 with elevated rice bran powder addition (*p* < 0.05), corresponding to reduced lightness and visually detectable darkening of cookies. This was attributed to the inherent brown pigments of rice bran. The a* value showed no significant difference among all groups. The b* value remained statistically unchanged at low rice bran powder addition levels, whereas a significant reduction occurred when the addition amount reached 14.00 g. These results demonstrate that rice bran powder exerted a negligible effect on redness yet significantly lowered the yellowness of cookies at higher addition levels.

For textural properties, the hardness of cookies decreased by 32.8% from 44.54 N to 29.91 N as the addition amount of rice bran powder increased from 6.00 g to 14.00 g (Table 4). The lowest values of cohesiveness, springiness, gumminess and chewiness were observed at the addition level of 12.00 g, with a slight and non-significant increase at 14.00 g. Overall, the hardness, cohesiveness, springiness, gumminess and chewiness of cookies all showed a decreasing trend with the increasing addition of rice bran powder, which was consistent with the previous findings that dietary fiber addition reduced all TPA parameters of baked cookies [41]. These textural variations can be primarily attributed to the complex compositional properties of rice bran. As a multi-component ingredient rich in dietary fiber, rice bran powder incorporates various non-starch components into the cookie matrix, thereby altering its structural properties. The dietary fiber fraction in rice bran physically disrupts the continuity of the gluten network, while the overall incorporation of rice bran dilutes the relative proportion of gluten protein in the dough. This interference inhibits the cross-linking and complete formation of continuous gluten structures, ultimately forming a looser, more porous internal texture in cookies and reducing their textural firmness and structural integrity [41].

Sensory scores of cookies increased first and then decreased with the increase in rice bran powder (Figure 1). The maximum scores were obtained at 8.00 g and 10.00 g addition, which were significantly higher than those of the 6.00 g, 12.00 g and 14.00 g groups (*p* < 0.05). Cookies with low rice bran addition exhibited excessive hardness and poor palatability. At the addition of 8.00 g and 10.00 g, the products possessed moderate crispness, uniform color and mild rice bran flavor, presenting the optimal sensory acceptability. When the addition exceeded 10.00 g, excessively dark color, rough mouthfeel and strong bran off-flavor led to a remarkable reduction in sensory scores. Detailed sensory evaluation data for each treatment are provided in Table S1.

Combining physicochemical, textural, and sensory results, the addition of 8.00 g and 10.00 g rice bran powder achieved a comprehensive balance in water activity, color, texture and sensory quality of cookies. Thus, the addition of 8.00 g or 10.00 g was identified as the optimal addition level, which effectively improved dietary fiber content without causing adverse effects on edible quality.

#### 3.1.2. Effect of Elderberry Powder Addition on Physicochemical Properties, Textural Properties, and Sensory Scores of Cookies

Elderberry powder is recognized as a natural functional ingredient rich in anthocyanins and phenolic compounds [10]. Incorporation of elderberry powder enhances nutritional value while imparting a characteristic pale pink color and distinctive flavor to cookies. However, excessive inclusion may adversely affect sensory quality due to the high dietary fiber content and intense flavor profile of elderberry powder [22].

As shown in Table 5, as the amount of elderberry powder increased, the *a_w_* values of the cookies decreased significantly from 0.539 to 0.432–0.434 (*p* < 0.05). This trend can be partly attributed to the inherently low water activity of dried elderberry powder itself. In addition, soluble dietary fiber, polysaccharides and other components contained in elderberry powder possess strong water-holding capacity [11]. Dietary fiber in fruit powders can effectively reduce the free water content in products by enhancing water binding and retention [42]. For color, the L* value of the cookies decreased markedly from 52.54 to 46.18 with increasing elderberry powder (*p* < 0.05), indicating reduced lightness. The a* value increased significantly from 6.27 to 10.99 (*p* < 0.05), while the b* value dropped from 19.56 to 13.14 (*p* < 0.05). The findings of the present study are basically consistent with those reported by Park et al., who found that the L* and b* values decreased significantly, while the a* value increased significantly with the increase in elderberry powder addition [12]. These results demonstrated that elderberry powder markedly increased redness and decreased yellowness of the cookies, which was directly associated with its abundant natural pigments such as anthocyanins. Similar color changes—decreased L* and b* values along with increased a* values—have been reported in cookies supplemented with maqui berry powder [43] and blackcurrant pomace powder [44], suggesting a consistent effect of dark berry powders on the color of baked products.

Regarding textural properties (Table 5), the hardness of cookies increased from 22.81 N to 40.94 N as the addition amount of elderberry powder rose from 1.00 g to 5.00 g, corresponding to a 79.5% increase. Cohesiveness decreased from 0.243 to 0.185, and springiness declined from 1.26 mm to 1.08 mm, showing an overall downward trend. Meanwhile, gumminess and chewiness increased significantly. These textural modifications stem from the characteristic chemical composition of elderberry powder. Previous studies have shown that elderberry fruit contains dietary fiber, pectin and other constituents [11], which competed with gluten proteins for free water and restricted full hydration and cross-linking of the gluten network. Meanwhile, the physical filling effect of insoluble particles disrupted the continuity of the gluten skeleton [45]. Furthermore, elderberry powder is rich in polyphenols, including anthocyanins and proanthocyanidins [11], which could reduce protein flexibility or alter the network structure by interacting with gluten proteins [46], further densifying the internal structure and weakening the skeleton continuity of the cookies. Consequently, the cookies showed higher hardness, gumminess and chewiness but lower cohesiveness and springiness.

**Figure 2:**
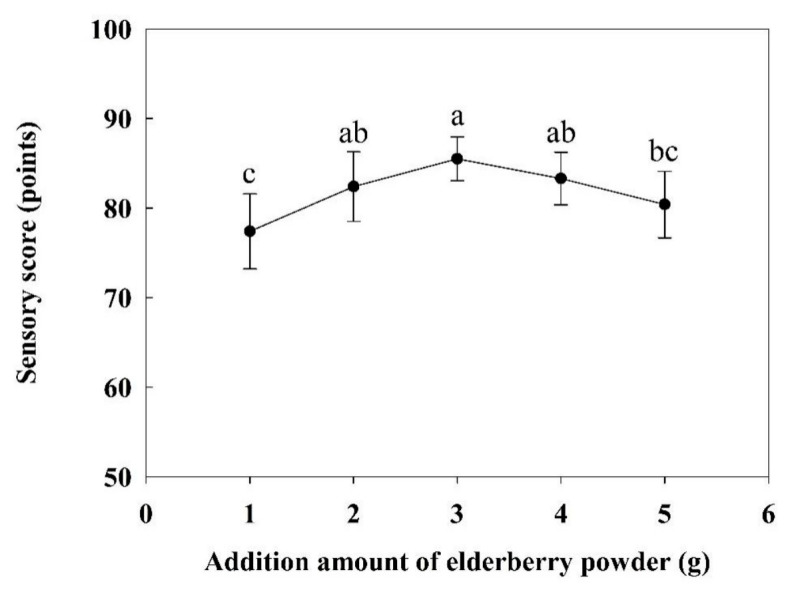
Effect of elderberry powder addition amount on the sensory score of cookies. Different lowercase letters indicate significant differences at *p* < 0.05.

The effect of elderberry powder addition on cookie sensory scores is shown in Figure 2. Sensory scores initially increased and then declined as the level of elderberry powder increased, reaching the highest score (85.5 points) at 3.00 g, which was significantly higher than that of the 1.00 g and 5.00 g groups (*p* < 0.05). At low addition levels, the cookies had weak flavor and inconspicuous color changes. At an elderberry powder addition of 3.00 g, the cookies showed uniform purplish-pink color, pleasant fresh elderberry aroma, and moderate crispness, achieving the best sensory acceptance. When the addition exceeded 3.00 g, the cookies became excessively dark, with intensified sourness and astringency of elderberry, as well as excessive hardness, leading to reduced palatability and lower sensory scores.

Based on the physicochemical, texture, and sensory results, the cookies achieved a balanced quality in water activity, color, texture and sensory properties at 3.00 g of elderberry powder, representing the optimal addition level. It endowed the products with natural color and flavor without impairing the edible quality.

#### 3.1.3. Effect of Butter Addition on Physicochemical Properties, Textural Properties, and Sensory Scores of Cookies

Butter is crucial in cookie formulation by influencing texture, flavor, and shelf stability [47]. Proper creaming of butter promotes the incorporation of air, which is essential for developing a porous structure and desirable crispness. The effect of butter addition on physicochemical and textural properties of cookies is shown in Table 6. With the increase in butter addition, the *a_w_* values of the cookies decreased significantly from 0.603 to 0.471 (*p* < 0.05). This was attributed to the reduced proportion of water in the formula system as butter content increased, along with the hydrophobic barrier formed by fat in the dough, which inhibited water migration and thus lowered water activity. This result is consistent with the findings of Milićević et al. [48] on the effect of fat replacers on the water activity of biscuits, which reported that full-fat biscuits had significantly lower water activity than low-fat biscuits, confirming the role of fat in reducing water activity. In terms of color, the L* value of cookies increased significantly from 50.99 to 55.36 (*p* < 0.05), and the b* value increased from 13.33 to 15.66 (*p* < 0.05) with rising butter addition, while the a* value showed a downward trend. These results indicated that butter addition improved the lightness and yellowness of cookies while reducing redness.

Regarding textural properties (Table 6), the hardness of cookies decreased significantly from 38.88 N to 23.06 N as the addition amount of butter increased from 60.00 g to 100.00 g (*p* < 0.05). Meanwhile, gumminess and chewiness also declined significantly from 6.52 N to 3.61 N and from 9.16 mJ to 4.03 mJ, respectively (*p* < 0.05). These changes were closely related to the “lubricating” and “diluting” effects of butter in the dough: butter coated gluten proteins, inhibiting excessive formation of the gluten network, and reduced starch gelatinization, resulting in a looser and more brittle texture, which is consistent with the regulatory mechanism of fat on cookie texture reported in previous studies [1]. Notably, cohesiveness, springiness, gumminess and chewiness all peaked in the 70.00 g butter group, which may be related to the dynamic balance achieved in the interactions between butter, gluten proteins and starch at this addition level, forming a texture with both moderate toughness and brittleness.

**Figure 3:**
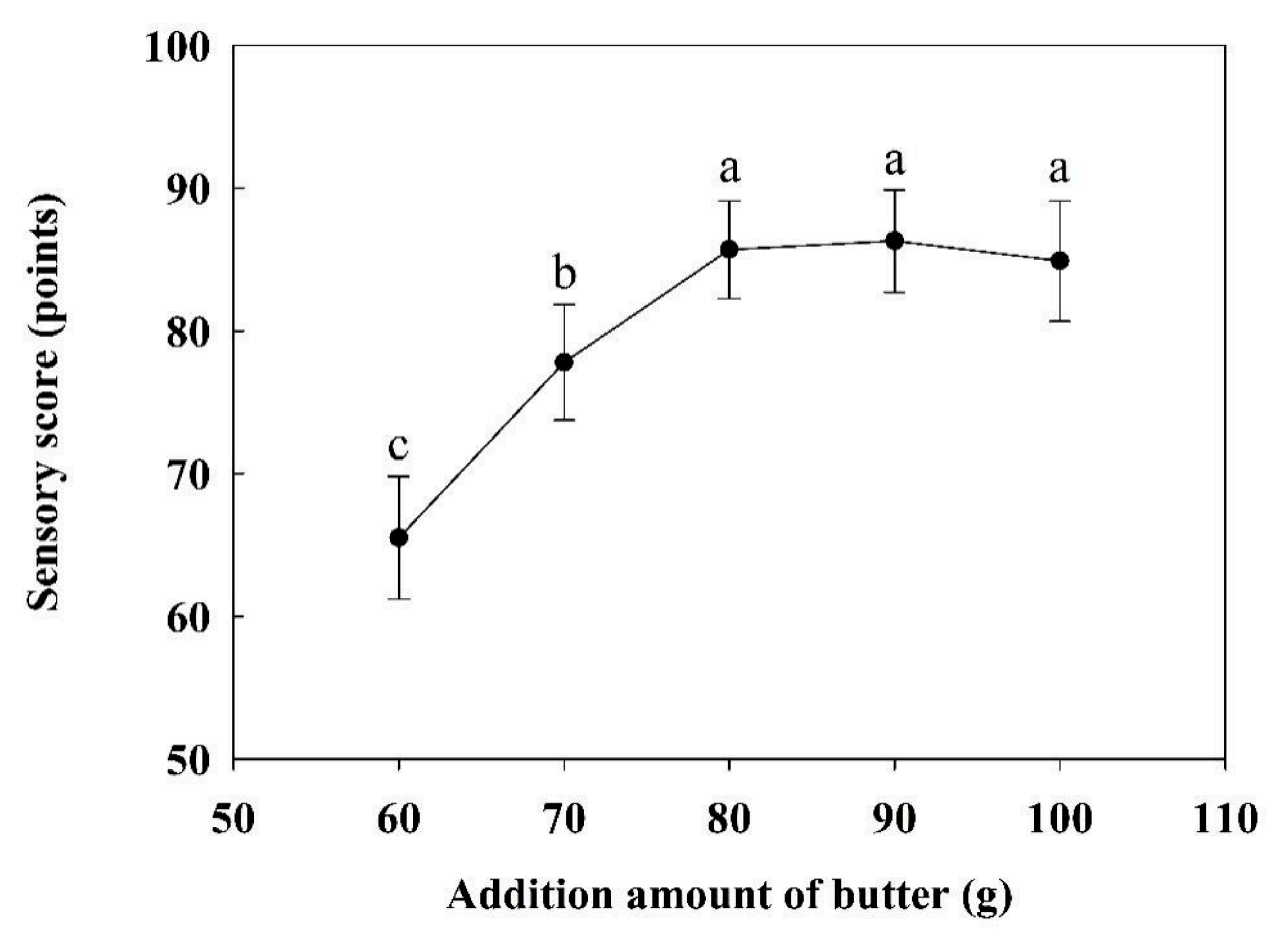
Effect of butter addition amount on the sensory score of cookies. Different lowercase letters indicate significant differences at *p* < 0.05.

Sensory scores of cookies first increased and then stabilized with increasing butter addition, reaching a plateau (>85 points) at 80.00–100.00 g, which was significantly higher than those of the 60.00 g and 70.00 g groups (*p* < 0.05) (Figure 3). At low butter addition levels, cookies were excessively hard and dry in mouthfeel and lacked butter flavor. At 80.00–100.00 g addition, the cookies exhibited moderate crispness, uniform golden color, and rich and balanced butter aroma, showing the optimal sensory acceptability. Excessive butter addition did not significantly improve sensory scores and may even lead to overly greasy taste and poor moldability.

Combining physicochemical, textural, and sensory results, the addition of 80.00 g and 90.00 g butter achieved a comprehensive balance in water activity, color, texture and sensory quality of cookies, which were determined as the optimal addition levels. These levels ensured the crisp mouthfeel and flavor of the cookies while avoiding sensory defects caused by excessive fat.

#### 3.1.4. Effect of Mogroside Addition on Physicochemical Properties, Textural Properties, and Sensory Scores of Cookies

Mogrosides, steviol glycosides, and FOS are widely used natural sweeteners that differ in sweetness intensity and physiological functionality. In the present study, these three sweeteners were combined to replace sucrose in cookie formulations. FOS and steviol glycosides were held at fixed levels of 2.000 g and 0.020 g, respectively, while the concentration of mogrosides was varied to identify an optimal dosage.

The effects of mogroside addition on physicochemical and textural properties of cookies are shown in Table 7. With the increase in mogroside addition, the *a_w_* values of the cookies showed a non-monotonic trend of first increasing and then decreasing, reaching a peak at the addition level of 0.300 g (*a_w_* = 0.509). This nonlinear response differed from the typical pattern observed with bulk sweetener replacement. In terms of color, the L* value of cookies decreased significantly from 53.52 to 47.95 as mogroside addition increased (*p* < 0.05). The b* value dropped significantly at an addition level of 0.200 g and then rebounded, while the a* value showed no significant difference among all groups. These results indicated that mogroside addition reduced the lightness of cookies and exerted certain effects on their yellowness.

For textural properties, the hardness of cookies exhibited a trend of first decreasing and then increasing, reaching the lowest value of 27.52 N at 0.200 g addition and then gradually rising to 40.77 N at 0.500 g (*p* < 0.05) (Table 7). Springiness showed an overall downward trend with increasing addition, with the lowest value of 0.94 mm observed in the 0.500 g group. Gumminess and chewiness also followed a similar pattern, decreasing first and then increasing, with the minimum values recorded at 0.200–0.300 g addition. Research on the application of mogrosides in cookies is still limited, and the specific mechanism underlying their effects on cookie properties requires further investigation.

**Figure 4:**
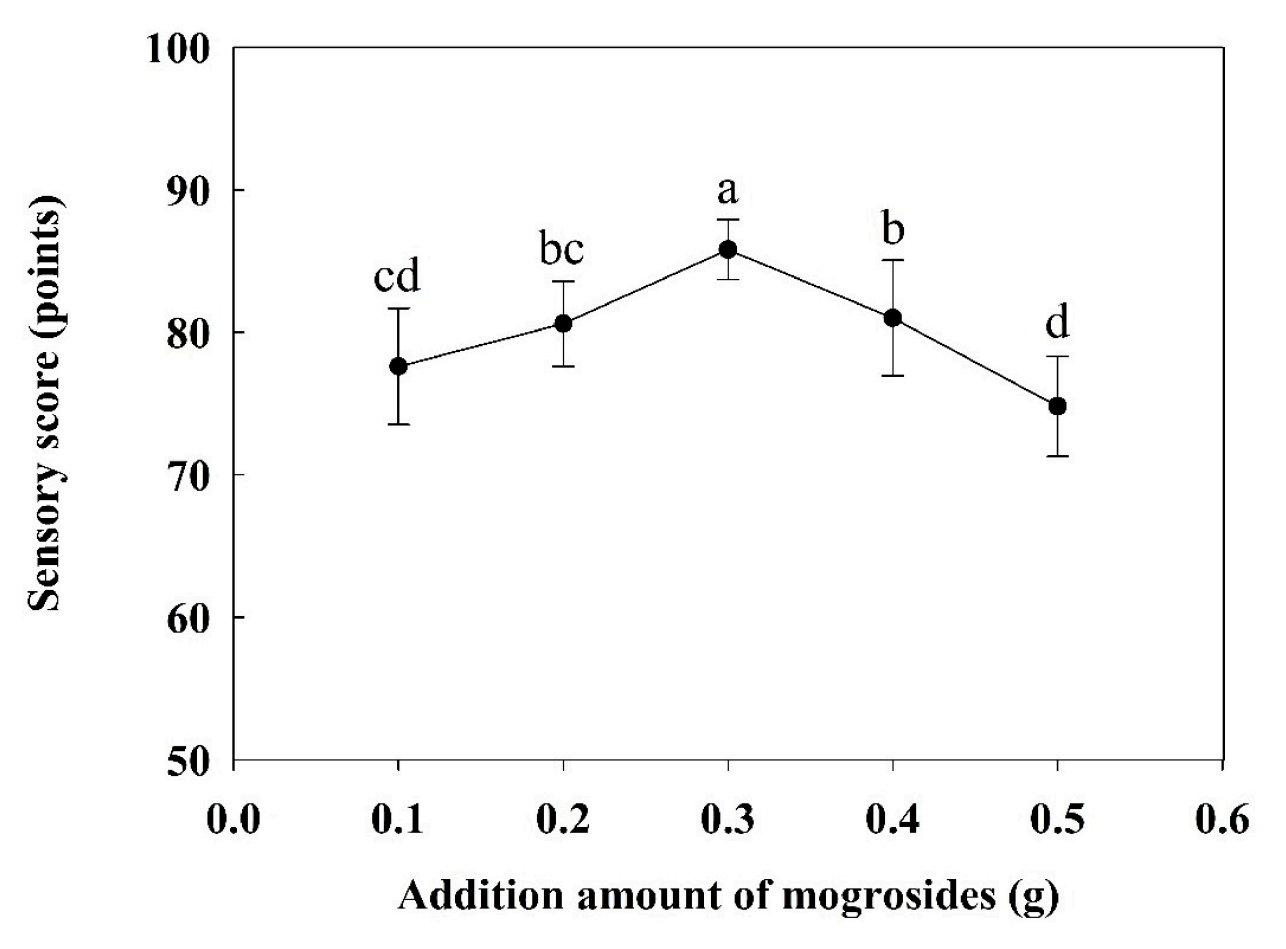
Effect of mogroside addition amount on the sensory quality of cookies. Different lowercase letters indicate significant differences at *p* < 0.05.

Sensory scores of cookies increased first and then decreased with increasing mogroside addition, peaking at the 0.300 g level (85.8 points), which was significantly higher than those of the 0.100 g and 0.500 g groups (*p* < 0.05, Figure 4). At low addition levels, cookies lacked sufficient sweetness and had a bland flavor. At 0.300 g addition, the cookies presented moderate sweetness, no obvious after-bitterness, and a crisp texture, showing the optimal sensory acceptability. When the addition exceeded 0.300 g, the characteristic after-bitterness of mogrosides gradually emerged, accompanied by increased hardness, which adversely affected palatability and led to a decline in sensory scores.

Combining physicochemical, textural, and sensory results, the addition of 0.300 g mogrosides achieved a comprehensive balance in water activity, color, texture and sensory quality of cookies. This level was determined as the optimal addition, which realized the no added sugar modification of the cookies without causing adverse effects on their edible quality.

### 3.2. Results of Response Surface Optimization Experiment

#### 3.2.1. Establishment of Response Surface Model and Analysis of Variance

Based on the Box–Behnken design, a total of 29 experimental runs were conducted, and the sensory scores for the cookie samples under different experimental conditions were recorded. The experimental matrix and observed response values are presented in Table 8. Quadratic polynomial regression analysis was performed using Design-Expert 13.0 to model the relationship between the sensory score (*Y*) and four independent variables: rice bran powder addition (*A*), elderberry powder addition (*B*), butter addition (*C*), and mogroside addition (*D*). The resulting second-order regression equation is as follows:

*Y* = 88.54 + 2.47*A* − 0.8500*B* + 0.3583*C* + 1.22*D* + 0.3250*AB* − 1.23*AC* − 0.6250*AD* − 2.98*BC* − 2.85*BD* − 0.4750*CD* − 5.77*A*^2^ − 5.26*B*^2^ − 2.97*C*^2^ − 6.61*D*^2^

The analysis of variance (ANOVA) for the regression model is summarized in Table 9. The results indicated that the model was highly significant (*p* < 0.0001), demonstrating that the regression equation accurately describes the relationship between the independent variables and the sensory score. The lack-of-fit test was not significant (*p* = 0.1416 > 0.05), suggesting no significant deviation between the experimental observations and model predictions and confirming the model’s adequacy. The coefficient of determination (*R*^2^) was 0.9870, indicating that 98.70% of the variation in sensory scores was attributable to the four independent variables, with only 1.30% due to random error. The adjusted *R*^2^ (*R*^2^*_Adj_*) was 0.9739, closely matching *R*^2^, further validating the reliability and predictive accuracy of the model.

The magnitude of the *F*-values reflects the relative contribution of each factor to the response. As shown in Table 9, the order of influence on sensory scores was: A (rice bran powder addition) > D (mogroside addition) > B (elderberry powder addition) > C (butter addition). Among the main factors, A, B, and D had highly significant effects on sensory scores (*p* < 0.01), whereas C was not significant (*p* = 0.1423 > 0.05). The dominant influence of rice bran powder (A) is attributed to its dual role in structure formation and flavor release, requiring precise control within a narrow range [21]. Mogrosides (D) exhibit high sweetness potency, making their dosage highly critical [18]. Elderberry powder (B) introduces polyphenol-derived bitterness that must be balanced with sweetness [49]. Butter (C) showed no significant effect, likely because the oil–sugar emulsion system was already stable [1]. Regarding interaction effects, AC, BC, and BD were highly significant (*p* < 0.01), while AB, AD, and CD were not significant (*p* > 0.05). The AC interaction (rice bran × butter) reflects the fat coating of fiber particles, which reduces roughness and modulates dough rheology. The BC interaction (elderberry × butter) involves hydrogen bonding between polyphenols and polar lipids, affecting fat distribution and masking bitterness. The BD interaction (elderberry × mogrosides) reveals a cross-modal synergy in which the floral/fruity aroma masks the licorice-like aftertaste of mogrosides [49]. All quadratic terms (A^2^, B^2^, C^2^, D^2^) were highly significant (*p* < 0.0001), indicating that the relationships between the independent variables and the response were nonlinear, with clear maxima on the response surface.

#### 3.2.2. Analysis of Two-Factor Interactions

**Figure 5:**
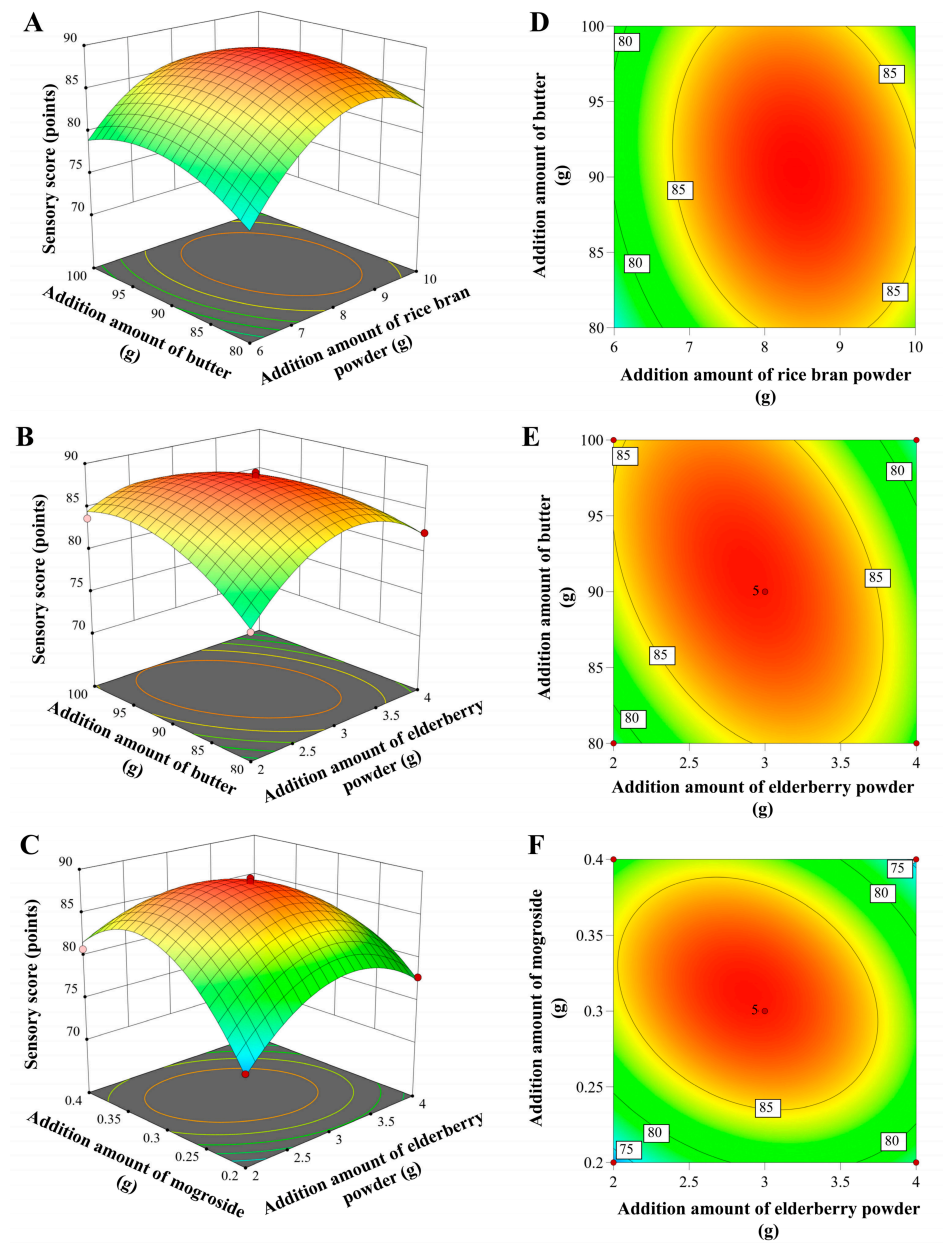
Response surface plots (**A**–**C**) and contour plots (**D**–**F**) for the interactive effects of rice bran powder and butter (**A**,**D**), elderberry powder and butter (**B**,**E**), and elderberry powder and mogrosides (**C**,**F**) on the sensory score of no added sugar cookies.

The response surface and contour plots depict the interactions between rice bran powder addition and butter addition, elderberry powder addition and butter addition, and elderberry powder addition and mogroside addition (Figure 5). The response surfaces show significant slopes across all three interactions, indicating that these factors exerted significant effects on the cookies’ sensory scores. Simultaneously, the contour plots for each interaction are elliptical, with the ellipse for the interaction between elderberry powder and butter showing the greatest elongation, which indicates that the collaborative matching of elderberry powder and butter played a decisive role in balancing sour taste and greasy perception, exerting the strongest influence on sensory quality. The results of the response surface and contour plot analyses were fully consistent with the ANOVA findings, further validating the significance of the interactions among the evaluated factors and confirming their critical roles in determining cookie sensory performance.

#### 3.2.3. Verification Experiment

The quadratic regression model was used to predict the optimal formulation for no added sugar cookies supplemented with rice bran powder and elderberry powder, using Design-Expert 13.0 software. The predicted optimum levels were 8.40 g rice bran powder, 2.88 g elderberry powder, 90.74 g butter, and 0.311 g mogrosides. For practical application, these values were rounded to 8 g rice bran powder, 3 g elderberry powder, 91 g butter, and 0.3 g mogrosides. The remaining ingredients were added according to the base recipe: 92.00 g low-gluten flour, 2.000 g FOS, 0.020 g steviol glycosides, 30.00 g beaten egg, and 20.00 g whole milk. Baking was carried out at 160 °C for 5 min to set the shape, followed by baking at 140 °C for 20–25 min to complete baking.

Verification was conducted through three parallel experiments using the optimized formulation. The cookies achieved an average sensory score of 89.0 ± 0.6 points, closely aligning with the model-predicted value of 88.5 points, with a relative error of 0.56%. These results indicate that the regression model is both reliable and accurate and that the optimized formulation is practical. The cookies had a uniform pale pink color, an intact shape, a crisp texture, and a harmonious balance of rice bran and elderberry aromas. The average weight of a single cookie was 8.28 ± 0.08 g, with an average diameter of 44.7 ± 0.4 mm and an average thickness of 9.2 ± 0.3 mm (mean ± SD, *n* = 10). Moreover, they were pleasantly sweet without excessive cloying, did not adhere to the teeth, and left no unpleasant aftertaste, demonstrating the effectiveness of the optimized formulation in achieving desirable sensory characteristics.

### 3.3. Nutritional Characteristics and Bioactive Compound Contents

#### 3.3.1. Nutritional Composition and Total Dietary Fiber Content

**Figure 6:**
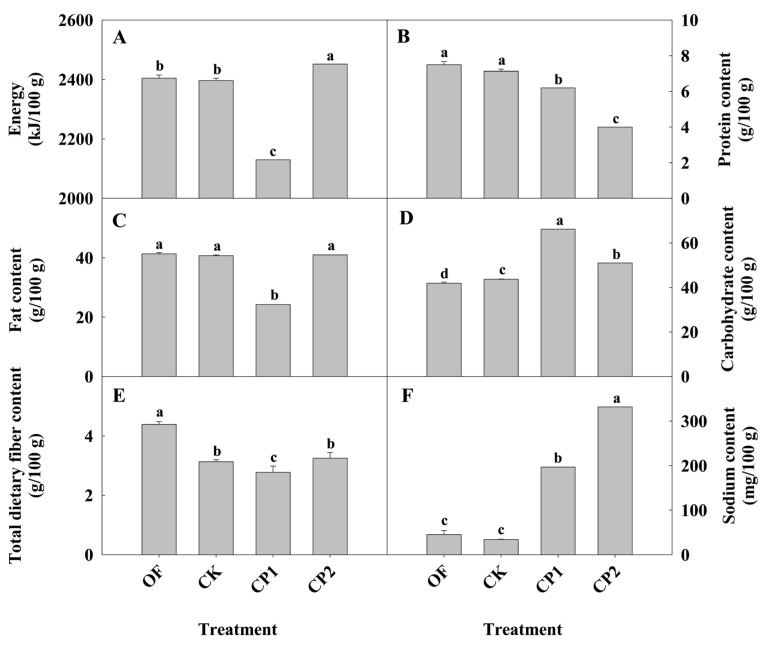
Nutritional composition and total dietary fiber content of the cookies. (**A**) Total energy (kJ/100 g); (**B**) protein content (g/100 g); (**C**) fat content (g/100 g); (**D**) carbohydrate content (g/100 g); (**E**) total dietary fiber content (g/100 g); (**F**) sodium content (mg/100 g). OF, the product obtained from the optimal formulation; CK, the control sample; CP1, commercial control product 1; CP2, commercial control product 2. Different lowercase letters indicate significant differences at the *p* < 0.05 level.

The nutritional composition of cookies prepared using the optimized formulation (OF) and the control sample (CK), as well as the total dietary fiber content of OF, CK, and two commercial cookie products (CP1 and CP2), was analyzed, and the results are presented in Figure 6. The ingredient lists of CP1 and CP2, as declared on their product labels, are provided in Section 2.1 (Materials); none of these commercial products contain rice bran powder, elderberry powder, or the ternary natural sweetener system used in OF. No significant differences were observed in energy, protein, fat, or sodium content between OF and CK. However, the carbohydrate content in OF was significantly lower than in CK (*p* < 0.05), whereas the total dietary fiber content was significantly higher (*p* < 0.05), increasing by 41.9%. These findings indicate that adding rice bran and elderberry powders effectively increased dietary fiber levels while reducing carbohydrate content. Compared with OF, CP1 had significantly lower fat content and energy value (*p* < 0.05). Based on the label ingredients of CP1, this difference likely reflects a lower proportion of fat in CP1, which also contributes to its firmer texture. However, because the complete formulation of CP1 is proprietary, the observed differences cannot be exclusively attributed to any single ingredient. No significant difference in fat content was observed between CP2 and OF. Protein and total dietary fiber contents in OF were significantly higher than in both CP1 (protein increased by 21.0%, total dietary fiber increased by 57.1%) and CP2 (protein increased by 87.5%, total dietary fiber increased by 33.3%) (*p* < 0.05). Moreover, OF contained significantly lower carbohydrate and sodium levels than CP1 (carbohydrate decreased by 36.7%, sodium decreased by 77.2%) and CP2 (carbohydrate decreased by 17.8%, sodium decreased by 86.4%) (*p* < 0.05). With the caveat that commercial product formulations are complex and proprietary, the results demonstrate that the no added sugar cookies supplemented with rice bran and elderberry powders have substantially higher protein and total dietary fiber content than the two tested commercial counterparts, indicating superior nutritional value within the context of this comparison. Simultaneously, the significant reductions in carbohydrate and sodium levels show additional health-promoting benefits [50], highlighting the potential of the optimized cookies as a nutritious and functional product for consumers seeking a balanced diet.

#### 3.3.2. Total Polyphenols, Flavonoids and Anthocyanins Content

**Figure 7:**
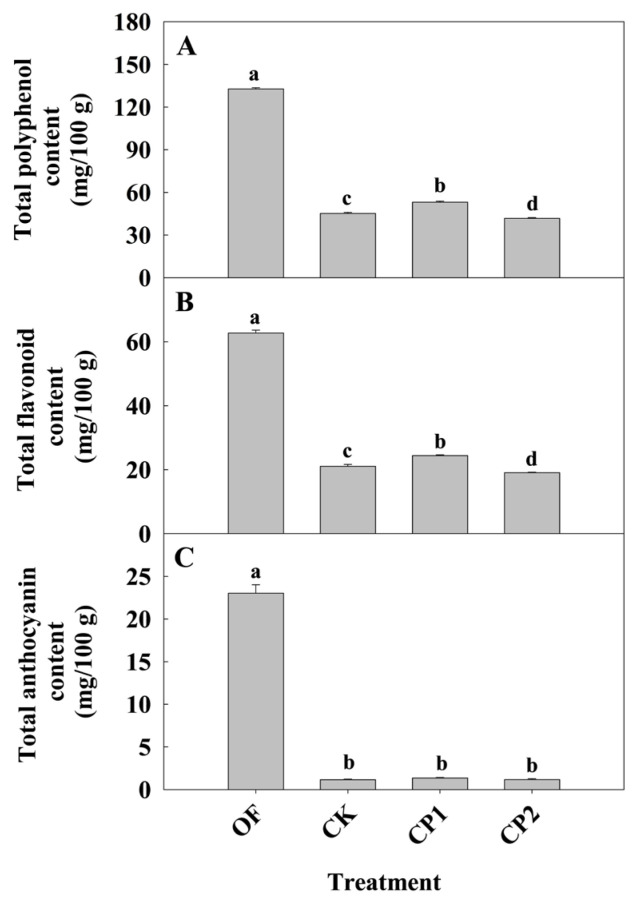
Contents of total polyphenols (**A**), flavonoids (**B**), and anthocyanins (**C**) in the cookies. OF, the product obtained from the optimal formulation; CK, the control sample; CP1, commercial control product 1; CP2, commercial control product 2. Different lowercase letters indicate significant differences at the *p* < 0.05 level.

As shown in Figure 7, the cookies prepared using the optimized formulation (OF) exhibited significantly higher total polyphenols (132.8 ± 0.85 mg/100 g), total flavonoids (62.77 ± 0.87 mg/100 g), and total anthocyanins (23.03 ± 0.99 mg/100 g) compared with the control sample (CK) and two commercial cookie products (CP1 and CP2). The elevated levels are mainly attributed to the inherent phenolic and flavonoid compounds in rice bran and elderberry powder as well as the anthocyanins from elderberry powder, despite partial thermal degradation during baking. In contrast, CK and CP2, made from refined wheat flour, contained only low background levels of these bioactive compounds. CP1 was supplemented with a small amount of dried coconut flesh and raisins, presenting higher contents of total polyphenols and total flavonoids than CK and CP2. Phenolic compounds, flavonoids, and anthocyanins are natural antioxidants that can scavenge free radicals, reduce inflammation, and help prevent chronic diseases; their contents are key indicators for evaluating the functional and health value of biscuits and other foods [51,52]. The results in this study indicate that the combined addition of rice bran and elderberry powder effectively enhances the functional quality of cookies.

## 4. Conclusions

This study developed no added sugar cookies supplemented with rice bran and elderberry powder using natural sweeteners (mogrosides, steviol glycosides, and FOS) as sucrose substitutes. Through single-factor experiments and Box–Behnken response surface methodology, the optimal formulation was determined as 8 g rice bran powder, 3 g elderberry powder, 91 g butter, and 0.3 g mogrosides, along with 92 g low-gluten flour, 2 g FOS, 0.02 g steviol glycosides, 30 g beaten egg, and 20 g milk, with a two-stage baking process (160 °C for 5 min, 140 °C for 20–25 min). The quadratic model was highly significant (*p* < 0.0001, *R*^2^ = 0.9870), with rice bran powder exerting the greatest influence on sensory quality. The optimized cookies exhibited a uniform pale pink color, an intact shape, a crisp texture, and a balanced flavor of rice bran and elderberry. Nutritional evaluation revealed that the optimized cookies had higher contents of protein, total dietary fiber, polyphenols, flavonoids, and anthocyanins, along with reduced carbohydrates and sodium relative to commercial cookie products, demonstrating an improved nutritional profile and promising potential functional value. This study facilitates the value-added utilization of rice bran and satisfies the growing consumer demand for healthy no added sugar baked goods. The findings provide theoretical and technical support for optimizing the formulation and potential industrial application of no added sugar functional cookies and may serve as a reference for developing other health-oriented food products. Future studies should focus on evaluating the shelf life and storage stability of the developed cookies. Further in vitro and in vivo studies on their antioxidant capacity and glycemic regulation effects are needed to validate their functional properties.

## Figures and Tables

**Table 1:** Basic recipe of no added sugar cookies (weight basis).

Raw Material	Weight (g)				
	Gradient 1	Gradient 2	Gradient 3	Gradient 4	Gradient 5
Low-Gluten Flour	94.00	92.00	90.00	88.00	86.00
Rice Bran Powder	6.00	8.00	10.00	12.00	14.00
Elderberry Powder	1.00	2.00	3.00	4.00	5.00
Butter	60.00	70.00	80.00	90.00	100.00
Mogrosides	0.100	0.200	0.300	0.400	0.500
Fructooligosaccharides	2.000	2.000	2.000	2.000	2.000
Steviol Glycosides	0.020	0.020	0.020	0.020	0.020
Beaten Egg	30.00	30.00	30.00	30.00	30.00
Whole Milk	20.00	20.00	20.00	20.00	20.00

Note: Low-Gluten Flour + Rice Bran Powder = 100.00 g; columns Gradient 1 to Gradient 5 correspond to five gradient levels set for each of the four tested variables: rice bran powder, elderberry powder, butter and mogrosides. Fructooligosaccharides, steviol glycosides, beaten egg and whole milk were added at constant dosages without adjustment in all formulations.

**Table 2:** Coded levels and corresponding actual values of the independent variables for the response surface optimization of the no added sugar cookie formulation.

Level	Rice Bran Powder (g)	Elderberry Powder (g)	Butter (g)	Mogrosides (g)
−1	6	2	80	0.2
0	8	3	90	0.3
1	10	4	100	0.4

**Table 3:** Sensory evaluation criteria for no added sugar cookies.

Indicator	Evaluation Standard	Sensory Score (Points)
Color (15 points)	Even color, glossy surface	11~15
	Relatively even color, faint gloss, slight surface scorching	6~10
	Uneven color, dull surface, noticeable scorching	0~5
Shape (15 points)	Intact shape, uniform thickness, no cracks, distinct surface, no shrinkage, no surface blistering	11~15
	Slightly incomplete shape, slight thickness inconsistency, minor surface cracks	6~10
	Severe incomplete shape, higher thickness inconsistency, no distinct surface, various cracks	0~5
Texture (20 points)	Easily broken, uniform internal structure, no large cracks, moderately crumbly	16~20
	Fairly easily broken, relatively uniform internal structure, a few cracks	6~15
	Difficult to break, uneven internal structure, many large cracks	0~5
Flavor (30 points)	Moderate and harmonious rice bran and elderberry aroma, sweet without cloying, no unpleasant aftertaste	21~30
	Too strong/faint rice bran and elderberry aroma, overly sweet/bland, slight unpleasant aftertaste	11~20
	Too intense/absent rice bran and elderberry aroma, bitter, or with unpleasant aftertaste, off-flavors	0~10
Mouthfeel (20 points)	Crisp, flaky texture; does not stick to teeth; not greasy; melts in the mouth	16~20
	Fairly crisp and flaky texture, slightly firm; does not stick to teeth or only slightly; slightly greasy	6~15
	Dense, hard, coarse, or excessively soft texture; sticks to teeth; greasy	0~5

**Table 4:** Effect of rice bran powder addition amount on the physicochemical and textural properties of cookies.

Addition Amount of Rice Bran Powder (g)	6.00	8.00	10.00	12.00	14.00
Water Activity (*a_w_*)	0.504 ± 0.008 ^a^	0.491 ± 0.004 ^a^	0.467 ± 0.015 ^b^	0.468 ± 0.014 ^b^	0.423 ± 0.007 ^c^
L*	51.26 ± 1.07 ^a^	51.46 ± 0.45 ^a^	47.85 ± 0.50 ^b^	43.60 ± 0.77 ^c^	42.83 ± 1.20 ^c^
a*	9.27 ± 0.25 ^a^	9.14 ± 0.24 ^a^	9.08 ± 0.56 ^a^	8.87 ± 0.41 ^a^	8.67 ± 0.33 ^a^
b*	16.41 ± 0.29 ^a^	16.52 ± 0.11 ^a^	16.49 ± 0.64 ^a^	15.94 ± 0.30 ^ab^	15.32 ± 0.49 ^b^
Hardness (N)	44.54 ± 0.93 ^a^	40.92 ± 1.87 ^b^	33.57 ± 1.42 ^c^	30.60 ± 1.56 ^d^	29.91 ± 1.53 ^d^
Cohesiveness	0.246 ± 0.022 ^a^	0.192 ± 0.008 ^b^	0.176 ± 0.013 ^b^	0.170 ± 0.005 ^b^	0.179 ± 0.002 ^b^
Springiness (mm)	1.16 ± 0.08 ^a^	1.07 ± 0.05 ^ab^	1.03 ± 0.04 ^bc^	0.96 ± 0.03 ^c^	1.04 ± 0.03 ^bc^
Gumminess (N)	10.97 ± 1.19 ^a^	7.86 ± 0.58 ^b^	5.88 ± 0.25 ^c^	5.20 ± 0.37 ^c^	5.35 ± 0.29 ^c^
Chewiness (mJ)	12.71 ± 1.32 ^a^	8.42 ± 0.37 ^b^	6.06 ± 0.02 ^c^	5.00 ± 0.22 ^c^	5.57 ± 0.40 ^c^

Note: Different lowercase letters in the same row indicate significant differences at *p* < 0.05.

**Table 5:** Effect of elderberry powder addition amount on the physicochemical and textural properties of cookies.

Addition Amount of Elderberry Powder (g)	1.00	2.00	3.00	4.00	5.00
Water Activity (*a_w_*)	0.539 ± 0.009 ^a^	0.514 ± 0.009 ^b^	0.474 ± 0.008 ^c^	0.432 ± 0.017 ^d^	0.434 ± 0.013 ^d^
L*	52.54 ± 1.25 ^a^	51.77 ± 0.69 ^a^	48.97 ± 0.89 ^b^	47.62 ± 0.25 ^bc^	46.18 ± 0.58 ^c^
a*	6.27 ± 0.50 ^d^	7.21 ± 0.41 ^c^	9.10 ± 0.33 ^b^	10.31 ± 0.20 ^a^	10.99 ± 0.40 ^a^
b*	19.56 ± 1.36 ^a^	16.66 ± 0.98 ^b^	15.14 ± 0.74 ^bc^	13.94 ± 1.66 ^c^	13.14 ± 0.38 ^c^
Hardness (N)	22.81 ± 1.26 ^d^	24.04 ± 0.70 ^d^	29.48 ± 1.24 ^c^	32.94 ± 1.20 ^b^	40.94 ± 1.06 ^a^
Cohesiveness	0.243 ± 0.012 ^a^	0.222 ± 0.008 ^b^	0.209 ± 0.010 ^bc^	0.200 ± 0.004 ^cd^	0.185 ± 0.010 ^d^
Springiness (mm)	1.26 ± 0.06 ^a^	1.17 ± 0.06 ^ab^	1.09 ± 0.07 ^b^	1.09 ± 0.01 ^b^	1.08 ± 0.01 ^b^
Gumminess (N)	5.53 ± 0.06 ^c^	5.34 ± 0.04 ^c^	6.18 ± 0.56 ^b^	6.59 ± 0.37 ^b^	7.55 ± 0.24 ^a^
Chewiness (mJ)	6.95 ± 0.40 ^bc^	6.27 ± 0.34 ^c^	6.71 ± 0.65 ^bc^	7.16 ± 0.44 ^b^	8.12 ± 0.33 ^a^

Note: Different lowercase letters in the same row indicate significant differences at *p* < 0.05.

**Table 6:** Effect of butter addition amount on the physicochemical and textural properties of cookies.

Addition Amount of Butter (g)	60.00	70.00	80.00	90.00	100.00
Water Activity (*a_w_*)	0.603 ± 0.010 ^a^	0.581 ± 0.012 ^b^	0.495 ± 0.011 ^c^	0.472 ± 0.009 ^d^	0.471 ± 0.004 ^d^
L*	50.99 ± 0.93 ^d^	52.23 ± 0.75 ^c^	52.84 ± 0.60 ^bc^	53.79 ± 0.52 ^b^	55.36 ± 0.43 ^a^
a*	9.40 ± 0.39 ^a^	9.13 ± 0.53 ^a^	8.33 ± 0.21 ^b^	8.52 ± 0.08 ^b^	8.47 ± 0.08 ^b^
b*	13.33 ± 0.72 ^b^	14.48 ± 0.33 ^a^	14.90 ± 0.73 ^a^	15.10 ± 0.67 ^a^	15.66 ± 0.57 ^a^
Hardness (N)	38.88 ± 1.70 ^a^	31.76 ± 1.85 ^b^	25.74 ± 0.81 ^c^	24.73 ± 0.70 ^cd^	23.06 ± 0.70 ^d^
Cohesiveness	0.168 ± 0.007 ^c^	0.231 ± 0.011 ^a^	0.184 ± 0.013 ^b^	0.166 ± 0.001 ^c^	0.157 ± 0.003 ^c^
Springiness (mm)	1.41 ± 0.10 ^b^	1.62 ± 0.05 ^a^	1.44 ± 0.05 ^b^	1.09 ± 0.03 ^c^	1.12 ± 0.00 ^c^
Gumminess (N)	6.52 ± 0.37 ^b^	7.36 ± 0.80 ^a^	4.73 ± 0.43 ^c^	4.12 ± 0.14 ^cd^	3.61 ± 0.06 ^d^
Chewiness (mJ)	9.16 ± 0.35 ^b^	11.96 ± 1.62 ^a^	6.78 ± 0.47 ^c^	4.47 ± 0.20 ^d^	4.03 ± 0.07 ^d^

Note: Different lowercase letters in the same row indicate significant differences at *p* < 0.05.

**Table 7:** Effect of mogroside addition amount on the physicochemical and textural properties of cookies.

Addition Amount of Mogrosides (g)	0.100	0.200	0.300	0.400	0.500
Water Activity (*a_w_*)	0.415 ± 0.018 ^c^	0.488 ± 0.026 ^ab^	0.509 ± 0.021 ^a^	0.458 ± 0.027 ^b^	0.360 ± 0.003 ^d^
L*	53.52 ± 0.71 ^a^	51.84 ± 0.78 ^b^	50.42 ± 0.69 ^c^	49.33 ± 0.94 ^c^	47.95 ± 0.44 ^d^
a*	9.12 ± 0.77 ^a^	8.98 ± 0.04 ^a^	8.69 ± 0.25 ^a^	8.61 ± 0.60 ^a^	8.79 ± 0.19 ^a^
b*	15.29 ± 0.72 ^a^	13.06 ± 0.16 ^c^	13.68 ± 0.32 ^bc^	14.35 ± 0.58 ^b^	15.44 ± 0.54 ^a^
Hardness (N)	35.88 ± 0.76 ^b^	27.52 ± 1.82 ^c^	29.36 ± 1.60 ^c^	37.04 ± 0.78 ^b^	40.77 ± 1.10 ^a^
Cohesiveness	0.252 ± 0.007 ^a^	0.237 ± 0.011 ^ab^	0.230 ± 0.008 ^ab^	0.228 ± 0.018 ^ab^	0.223 ± 0.015 ^b^
Springiness (mm)	1.11 ± 0.04 ^a^	1.08 ± 0.09 ^a^	1.09 ± 0.06 ^a^	1.00 ± 0.04 ^ab^	0.94 ± 0.03 ^b^
Gumminess (N)	9.03 ± 0.39 ^a^	6.52 ± 0.17 ^b^	6.77 ± 0.61 ^b^	8.45 ± 0.73 ^a^	9.08 ± 0.66 ^a^
Chewiness (mJ)	10.01 ± 0.62 ^a^	7.05 ± 0.73 ^c^	7.35 ± 0.32 ^c^	8.48 ± 0.76 ^b^	8.53 ± 0.46 ^b^

Note: Different lowercase letters in the same row indicate significant differences at *p* < 0.05.

**Table 8:** Box–Behnken response surface design and sensory scores.

No.	Rice Bran Powder (A)	Elderberry Powder (B)	Butter (C)	Mogrosides (D)	Sensory Score (Points)
1	0	0	−1	1	80.9
2	0	−1	−1	0	77.5
3	−1	0	−1	0	75.5
4	0	−1	0	1	80.8
5	1	0	0	1	79.8
6	0	0	0	0	87.8
7	0	1	−1	0	82.3
8	−1	0	0	−1	70.7
9	0	−1	1	0	83.7
10	1	0	1	0	81.2
11	0	0	0	0	88.8
12	0	0	0	0	88.3
13	0	−1	0	−1	73.7
14	1	−1	0	0	81.4
15	−1	0	1	0	79.4
16	0	0	−1	−1	77.6
17	1	0	0	−1	77.5
18	0	0	1	1	80.4
19	0	0	1	−1	79
20	0	1	0	−1	77.8
21	−1	−1	0	0	77
22	0	0	0	0	88.7
23	1	0	−1	0	82.2
24	1	1	0	0	79.7
25	0	0	0	0	89.1
26	−1	0	0	1	75.5
27	−1	1	0	0	74
28	0	1	0	1	73.5
29	0	1	1	0	76.6

**Table 9:** Analysis of variance for the regression model.

Source of Variation	Sum of Squares	Degrees of Freedom	Mean Square	*F*-Value	*p*-Value	Significance
Model	675.65	14	48.26	75.72	<0.0001	significant
A-A	73.51	1	73.51	115.33	<0.0001	**
B-B	8.67	1	8.67	13.60	0.0024	**
C-C	1.54	1	1.54	2.42	0.1423	
D-D	17.76	1	17.76	27.87	0.0001	**
AB	0.4225	1	0.4225	0.6629	0.4292	
AC	6.00	1	6.00	9.42	0.0083	**
AD	1.56	1	1.56	2.45	0.1397	
BC	35.40	1	35.40	55.55	<0.0001	**
BD	32.49	1	32.49	50.98	<0.0001	**
CD	0.9025	1	0.9025	1.42	0.2538	
A^2^	215.95	1	215.95	338.83	<0.0001	**
B^2^	179.29	1	179.29	281.32	<0.0001	**
C^2^	57.22	1	57.22	89.77	<0.0001	**
D^2^	283.19	1	283.19	444.33	<0.0001	**
Residual	8.92	14	0.6373			
Lack of Fit	7.91	10	0.7911	3.13	0.1416	not significant
Pure Error	1.01	4	0.2530			
Total	684.57	28				

Note: ** indicates extremely significant (*p* < 0.01).

## Data Availability

The data presented in this study are available on request from the corresponding author due to privacy or ethical restrictions.

## Supplementary Materials

The following supporting information can be downloaded at: https://www.mdpi.com/article/10.3390/foods15152584/s1, Table S1. Sensory evaluation scores of samples with different addition levels of rice bran powder, elderberry powder, butter and mogrosides.

## References

[1] Devi A., Khatkar B. (2016). Physicochemical, rheological and functional properties of fats and oils in relation to cookie quality: A review. J. Food Sci. Technol.-Mysore.

[2] Suo X., Tagliasco M., Bonfini M., Bonfili L., Araiza O.M., Baggio A., Eleuteri A.M., Pellegrini N., Vittadini E. (2025). Development of sugar- and fat-reduced pulse cookies with improved predicted glycemic behavior. Appl. Food Res..

[3] Tan B., Norhaizan M., Chan L. (2023). Rice bran: From waste to nutritious food ingredients. Nutrients.

[4] Kodape A., Kodape A., Desai R. (2025). Rice bran: Nutritional value, health benefits, and global implications for aflatoxin mitigation, cancer, diabetes, and diarrhea prevention. Food Chem..

[5] Gu I., Lam W., Marasini D., Brownmiller C., Savary B., Lee J., Carbonero F., Lee S. (2021). In vitro fecal fermentation patterns of arabinoxylan from rice bran on fecal microbiota from normal-weight and overweight/obese subjects. Nutrients.

[6] Mahalak K., Liu L., Bobokalonov J., Narrowe A., Firrman J., Bittinger K., Hu W., Jones S., Moustafa A. (2024). Supplementation with soluble or insoluble rice-bran fibers increases short-chain fatty acid producing bacteria in the gut microbiota in vitro. Front. Nutr..

[7] Saji N., Francis N., Schwarz L.J., Blanchard C.L., Santhakumar A.B. (2020). The antioxidant and anti-inflammatory properties of rice bran phenolic extracts. Foods.

[8] Kalita P., Ahmad A.B., Sen S., Deka B., Hazarika Q.K., Kapil M.J., Pachuau L. (2023). High value compounds and bioactivity of rice bran, rice bran protein: A review. Recent Adv. Food Nutr. Agric..

[9] Ronie M., Mamat H., Aziz A., Sarjadi M., Mokhtar R., Putra N. (2024). Rice bran as a potent ingredient: Unveiling its potential for value-added applications. Food Sci. Biotechnol..

[10] Sanlier N., Ejder Z.B., Irmak E. (2024). Are the effects of bioactive components on human health a myth?: Black elderberry (*Sambucus nigra* L.) from exotic fruits. Curr. Nutr. Rep..

[11] Młynarczyk K., Walkowiak-Tomczak D., Łysiak G.P. (2018). Bioactive properties of *Sambucus nigra* L. as a functional ingredient for food and pharmaceutical industry. J. Funct. Foods.

[12] Park M.-H., Kim D.S., Kim M. (2025). Quality and antioxidant properties of wheat cookies supplemented with elderberry powder. Culin. Sci. Hosp. Res..

[13] Pareyt B., Delcour J.A. (2008). The role of wheat flour constituents, sugar, and fat in low moisture cereal based products: A review on sugar-snap cookies. Crit. Rev. Food Sci. Nutr..

[14] World Health Organization (2015). Sugars Intake for Adults and Children.

[15] Bali V., Panesar P., Bera M., Panesar R. (2015). Fructo-oligosaccharides: Production, purification and potential applications. Crit. Rev. Food Sci. Nutr..

[16] Seki H., Tamura K., Muranaka T. (2018). Plant-derived isoprenoid sweeteners: Recent progress in biosynthetic gene discovery and perspectives on microbial production. Biosci. Biotechnol. Biochem..

[17] Ceunen S., Geuns J. (2013). Steviol glycosides: Chemical diversity, metabolism, and function. J. Nat. Prod..

[18] Guo Q., Shi M., Sarengaowa, Xiao Z., Xiao Y., Feng K. (2024). Recent advances in the distribution, chemical composition, health benefits, and application of the fruit of *Siraitia grosvenorii*. Foods.

[19] Chen N., Cao W., Yuan Y., Wang Y., Zhang X., Chen Y., Yiasmin M., Tristanto N., Hua X. (2024). Recent advancements in mogrosides: A review on biological activities, synthetic biology, and applications in the food industry. Food Chem..

[20] Lv S., Sun L., Zhao S., Bao Y. (2017). Effect of dry heat stabilisation on the functional properties of rice bran proteins. Int. J. Food Sci. Technol..

[21] He J., Li H., Pi C., Tang Z., Xu J., Su W., Zhong S. (2022). Optimization of processing technology and quality characteristics of seawater rice bran biscuits. Sci. Technol. Cereals Oils Foods.

[22] Hlavácová Z., Ivanigsová E., Harangozo L., Petrovic A., Kusteková D., Gálik B., Hlavác P., Boziková M., Vozárová V. (2021). Physico-chemical and sensory profiles of enriched linz biscuits. Foods.

[23] Chai Y., Ping L., Wu D., Chang X. (2024). Formula optimization of low-calorie rice bran green tea cookies. Cereal Feed Ind..

[24] Heymann H., Machado B., Torri L., Robinson A. (2012). How many judges should one use for sensory descriptive analysis?. J. Sens. Stud..

[25] Lawless H.T., Heymann H. (2020). Sensory Evaluation of Food: Principles and Practices.

[26] (2023). Sensory Analysis—Selection and Training of Sensory Assessors.

[27] (2021). Sensory Analysis—Methodology—Guidelines for Assessing the Performance of a Quantitative Descriptive Sensory Panel.

[28] Xiao H., Zeng B., Gong Y., Liang Y., Xie J., Ye X., Zhan L., Tian Z. (2024). Development of *Ganoderma lucidum* cookies. Farm Prod. Process..

[29] (2011). National Food Safety Standard General Rules for Nutrition Labeling of Prepackaged Foods.

[30] (2016). National Food Safety Standard Determination of Protein in Food.

[31] (2016). National Food Safety Standard Determination of Fat in Food.

[32] (2017). National Food Safety Standard Determination of Sodium in Food.

[33] (2016). National Food Safety Standard Determination of Moisture in Food.

[34] (2016). National Food Safety Standard Determination of Ash in Food.

[35] (2023). National Food Safety Standard Determination of Total Dietary Fiber in Food.

[36] Wan L., Wang X., Zhao X., Zhao J., Zheng X., Xia Y., Peng L., Xiang D., Zou L., Jiang L. (2025). Processing technology and quality evaluation of compound biscuits with fermented *Gastrodia elata*. J. Agric. Livest. Prod. Process..

[37] (2016). Determination of Total Flavonoids in Export Food.

[38] Xing Y., Hui X., Sun S., Wang T., Sui Y. (2022). Effects of antioxidants on anthocyanins in purple sweet potato biscuits. China Fruit Veget..

[39] Zhao G., Zhang R., Dong L., Huang F., Tang X., Wei Z., Zhang M. (2018). Particle size of insoluble dietary fiber from rice bran affects its phenolic profile, bioaccessibility and functional properties. LWT.

[40] Abdul-Hamid A., Luan Y.S. (2000). Functional properties of dietary fibre prepared from defatted rice bran. Food Chem..

[41] Jia M., Yu Q., Chen J., He Z., Chen Y., Xie J., Nie S., Xie M. (2020). Physical quality and in vitro starch digestibility of biscuits as affected by addition of soluble dietary fiber from defatted rice bran. Food Hydrocoll..

[42] Salehi F., Aghajanzadeh S. (2020). Effect of dried fruits and vegetables powder on cakes quality: A review. Trends Food Sci. Technol..

[43] Jeon H.J., Lee J.-H. (2021). Quality and antioxidant properties of wheat cookies supplemented with maqui berry powder. Food Sci. Preserv..

[44] Nour V., Blejan A., Codina G. (2025). Use of bilberry and blackcurrant pomace powders as functional ingredients in cookies. Appl. Sci..

[45] Saric B., Dapcevic-Hadnadev T., Hadnadev M., Sakac M., Mandic A., Misan A., Skrobot D. (2019). Fiber concentrates from raspberry and blueberry pomace in gluten-free cookie formulation: Effect on dough rheology and cookie baking properties. J. Texture Stud..

[46] Girard A., Awika J. (2020). Effects of edible plant polyphenols on gluten protein functionality and potential applications of polyphenol-gluten interactions. Compr. Rev. Food Sci. Food Saf..

[47] Manley D. (2000). Technology of Biscuits, Crackers and Cookies.

[48] Milićević N., Sakač M., Hadnađev M., Škrobot D., Šarić B., Hadnađev T.D., Jovanov P., Pezo L. (2020). Physico-chemical properties of low-fat cookies containing wheat and oat bran gels as fat replacers. J. Cereal Sci..

[49] Pedziwiatr D., Lamadrid M., Wojdylo A. (2024). Cookies fortified with polyphenols extracts: Impact on phenolic content, antioxidant activity, inhibition of α-amylase and α-glucosidase enzyme, colour and sensory attractiveness. Antioxidants.

[50] Aishah B., Fadhilah J., Noriham A., Noorlaila A., Aisyah Jacklin L., Yun Irma F.E. (2020). Reformulation of Le’Natura^®^ biscuit: Effects on textural, sensorial, nutritional and glycemic index values. Int. Pharm. Acta.

[51] Bendokas V., Skemiene K., Trumbeckaite S., Stanys V., Passamonti S., Borutaite V., Liobikas J. (2020). Anthocyanins: From plant pigments to health benefits at mitochondrial level. Crit. Rev. Food Sci. Nutr..

[52] Sun W., Shahrajabian M. (2023). Therapeutic potential of phenolic compounds in medicinal plants-natural health products for human health. Molecules.

