# Supplementary material for: Formulation Optimization of No Added Sugar Cookies Supplemented with Rice Bran Powder and Elderberry Powder
Source: Foods. 2026 Jul 23;15(15):2584. doi: 10.3390/foods15152584 (PMC13464673; doi:10.3390/foods15152584)
Supplement: Supplementary file 1 [file foods-15-02584-s001.zip › foods-4408718-supplementary.pdf]

Table S1. Sensory evaluation scores of samples with different addition levels of rice bran powder, elderberry powder, butter and mogrosides.

Variables	Addition amount (g)	Color	Shape	Texture	Flavor	Mouthfeel	Total
Rice bran powder	6.00	12.1±0.6^{b}	13.3±0.8^{ab}	16.6±0.7^{bc}	18.2±1.4^{c}	14.9±2.5^{b}	75.0±4.1^{b}
	8.00	13.3±0.9^{a}	14.1±1.0^{a}	17.1±0.7^{ab}	24.4±2.0^{a}	17.4±0.9^{a}	86.2±4.2^{a}
	10.00	13.0±1.0^{a}	13.6±0.6^{a}	17.6±0.8^{a}	24.5±1.5^{a}	17.2±0.8^{a}	85.7±2.9^{a}
	12.00	10.3±0.5^{c}	12.7±1.5^{bc}	16.7±1.1^{bc}	21.1±1.7^{b}	15.6±1.0^{b}	76.3±4.0^{b}
	14.00	9.0±0.4^{d}	12.1±0.7^{c}	16.1±0.8^{c}	18.3±2.3^{c}	14.3±1.0^{b}	69.7±3.6^{c}
	p -value	<0.0001	0.0003	0.0046	<0.0001	<0.0001	<0.0001
Elderberry powder	1.00	12.4±0.7^{ab}	12.4±1.2^{a}	15.4±1.5^{b}	22.3±1.4^{c}	15.0±1.4^{c}	77.4±4.2^{c}
	2.00	12.9±0.8^{ab}	12.4±0.7^{a}	16.2±1.9^{ab}	24.5±1.6^{ab}	16.5±1.5^{ab}	82.4±3.9^{ab}
	3.00	13.1±0.8^{a}	12.8±0.5^{a}	16.8±1.1^{a}	25.6±0.8^{a}	17.2±0.6^{a}	85.5±2.5^{a}
	4.00	12.7±0.7^{ab}	12.9±0.5^{a}	16.7±0.9^{a}	24.3±1.6^{ab}	16.8±0.8^{a}	83.3±2.9^{ab}
	5.00	12.3±0.6^{b}	12.5±0.7^{a}	16.3±0.5^{ab}	23.8±1.6^{b}	15.5±1.6^{bc}	80.4±3.7^{bc}
	p -value	0.1013	0.4118	0.1149	0.0002	0.0009	<0.0001
Butter	60.00	10.2±0.8^{c}	11.3±1.3^{c}	12.4±0.7^{c}	22.1±1.3^{c}	9.7±1.0^{c}	65.5±4.3^{c}
	70.00	12.3±1.3^{b}	12.5±1.4^{b}	16.0±0.8^{b}	23.5±1.2^{b}	13.6±1.2^{b}	77.8±4.1^{b}
	80.00	13.1±1.3^{ab}	13.4±0.8^{ab}	16.9±0.7^{a}	25.3±1.1^{a}	17.2±0.9^{a}	85.7±3.4^{a}
	90.00	13.6±0.9^{a}	13.6±0.8^{a}	17.2±1.2^{a}	24.7±1.7^{ab}	17.3±0.7^{a}	86.3±3.6^{a}
	100.00	13.4±1.3^{a}	13.5±0.5^{a}	17.4±0.9^{a}	23.4±1.8^{b}	17.2±1.0^{a}	84.9±4.2^{a}
	p -value	<0.0001	<0.0001	<0.0001	<0.0001	<0.0001	<0.0001
Mogrosides	0.100	12.6±0.8^{ab}	12.0±0.7^{a}	15.9±0.7^{b}	21.5±2.7^{c}	15.7±1.2^{bc}	77.6±4.1^{cd}
	0.200	12.7±0.7^{ab}	12.2±1.0^{a}	16.4±0.6^{ab}	22.3±1.6^{bc}	17.0±1.1^{a}	80.6±3.0^{bc}
	0.300	13.2±0.9^{a}	12.5±0.5^{a}	16.7±0.3^{a}	25.8±1.2^{a}	17.7±0.8^{a}	85.8±2.1^{a}
	0.400	12.5±0.6^{ab}	12.2±2.1^{a}	16.1±0.7^{ab}	24.2±1.5^{ab}	16.0±0.7^{b}	81.0±4.1^{b}
	0.500	12.1±0.6^{b}	12.2±0.9^{a}	16.4±1.0^{ab}	19.3±3.9^{d}	15.0±0.6^{c}	74.8±3.5^{d}
	p -value	0.0366	0.8855	0.1092	<0.0001	<0.0001	<0.0001

Note: Different lowercase letters in the same column indicate significant differences at $p < 0.05$.
